# Supplementary material for: On the similarities of representations in artificial and brain neural networks for speech recognition
Source: Front Comput Neurosci. 2022 Dec 21;16:1057439. doi: 10.3389/fncom.2022.1057439 (PMC9811675; doi:10.3389/fncom.2022.1057439)
Supplement: Supplementary file 1 [file Data_Sheet_1.PDF]

Supplementary Material

SUPPLEMENTARY FIGURE

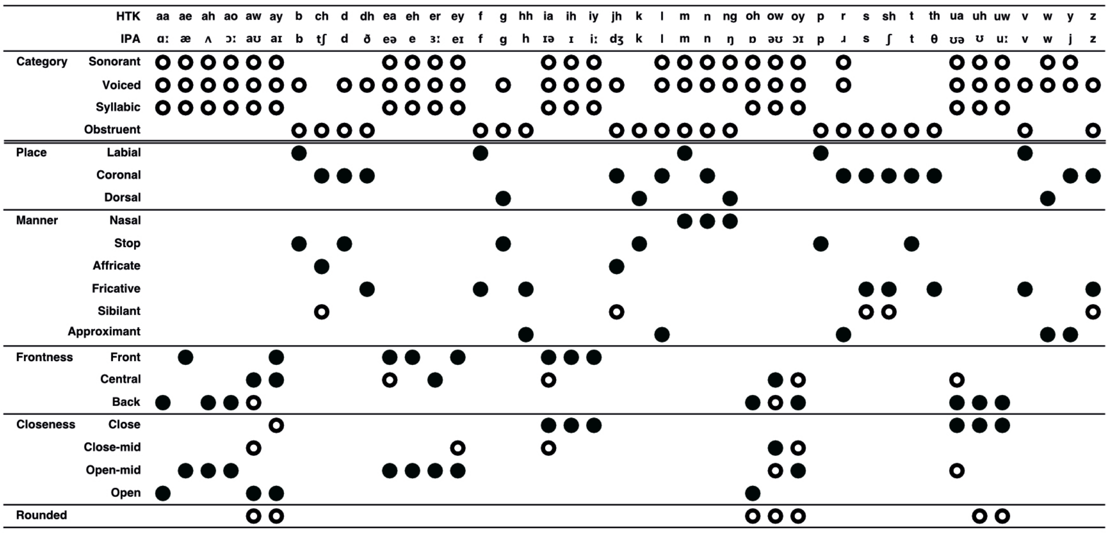

Figure S1. Phone–feature matrix: assignment of features to phones. Empty circles indicate presence of the feature for a phone. Where a phone has more than one feature for a given category, full circles indicate the dominant feature, used in clustering analysis.

SUPPLEMENTARY TABLE

Left hemisphere

DNN layer model	Cluster latency (ms) Start	Max	End	Peak extent (vertices)
FBK (early cluster)	0	20	70	93
FBK (late cluster)	150	180	200	100
L2	0	160	230	270
L3	0	170	250	237
L4	140	170	200	43
L5			(n.s.)	
L6	20	180	230	151
L7	40	170	230	129
TRI			(n.s.)	

Right hemisphere

DNN layer model	Cluster latency (ms) Start	Max	End	Peak extent (vertices)
FBK	0	170	120	186
L2	0	70	110	172
L3	0	0	110	341
L4	0	0	110	411
L5	0	50	70	20
L6	0	50	120	264
L7	0	40	120	286
TRI			(n.s.)	

Table S1. Clusters of fit for DNN-BN$_7$ in EMEG study. Latencies for left- and right-hemisphere clusters ($p < 0.01$) for each hidden-layer model.

SUPPLEMENTARY FIGURE

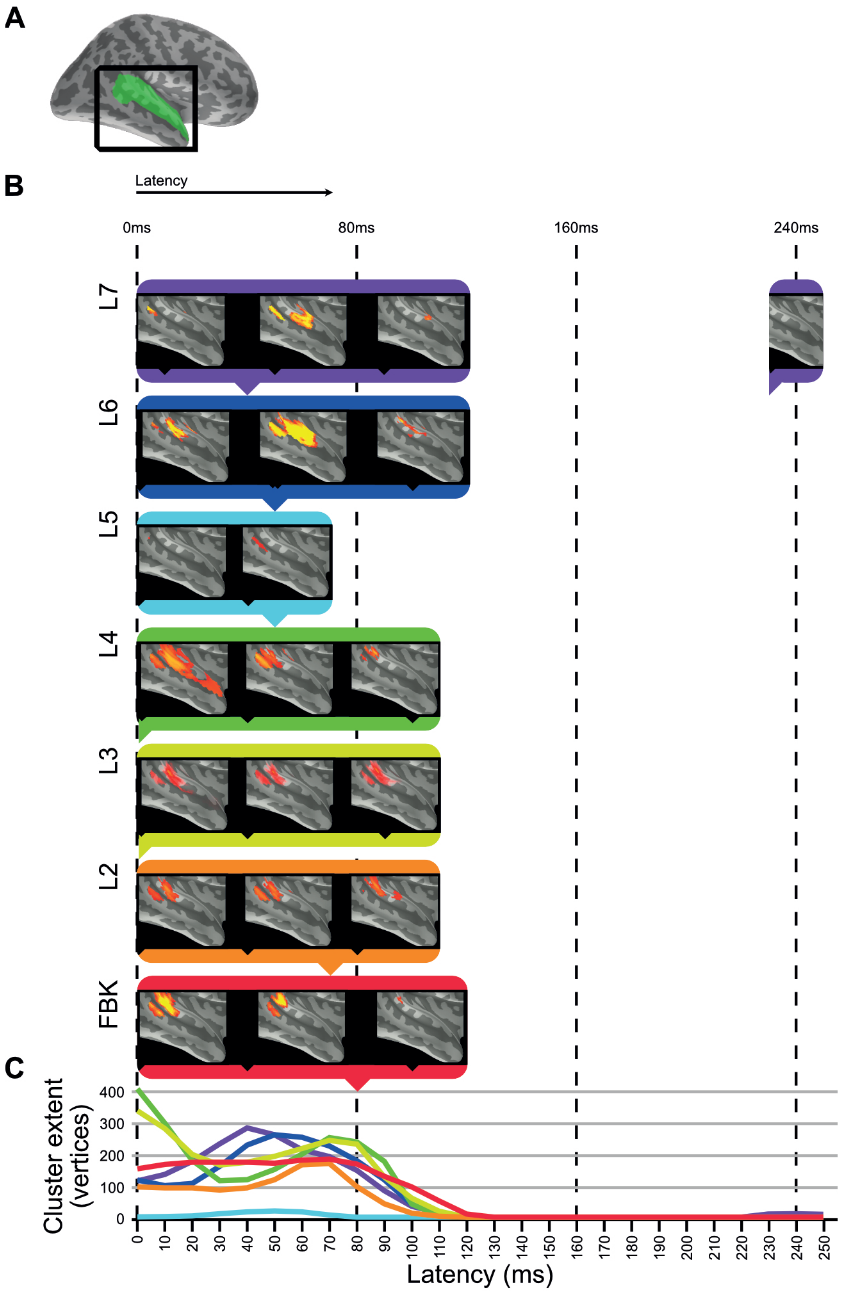

Figure S2. Clusters of significant fit of hidden-layer models to right-hemisphere EMEG data. (a) Location of region of interest mask for auditory cortex. **(b)** Maps describing fit of DNN layer models to EMEG data. All maps thresholded at $p < 0.01$ (corrected). **(c)** Line graphs showing the time-courses of cluster extents for each layer which showed significant fit.
